# Assessment of the Effects of Electromagnetic Fields on Apoptosis and Stress Protein Biomarkers in the Spider *Parasteatoda tepidariorum*

**DOI:** 10.3390/ijms27094088

**Published:** 2026-05-02

**Authors:** Weronika Porc, Katarzyna Rozpędek, Mateusz Glenszczyk, Artur Lis, Agnieszka Babczyńska

**Affiliations:** Faculty of Natural Sciences, University of Silesia in Katowice, Bankowa 9, 40-007 Katowice, Poland; weronika.porc@us.edu.pl (W.P.); katarzyna.rozpedek@us.edu.pl (K.R.); mateusz.glenszczyk@us.edu.pl (M.G.); artur.lis@us.edu.pl (A.L.)

**Keywords:** heat shock proteins, egg cocoon, apoptosis, developmental stages, electromagnetic fields (EMFs)

## Abstract

Electromagnetic fields (EMFs), increasingly prevalent due to technological advancements, have raised significant concerns regarding their potential biological effects on living organisms. While much attention has focused on human health, growing evidence suggests that EMFs can also affect invertebrates, which play vital ecological roles. This study investigates the biochemical and cell death biomarker responses to EMF exposure for 24 h or 72 h in *Parasteatoda tepidariorum*. The focus is placed on the 10 MHz frequency, which is relevant to environmental exposure scenarios. Biochemical biomarkers include heat shock proteins (HSP70) and the percentage of apoptotic and living cells in individuals at their embryonic, young and adult stages. Results indicate that exposure to EMFs can induce measurable stress responses at the biochemical level, with variations depending on developmental stage and protective structures. Embryos outside of the egg sac exhibited significantly elevated levels of HSP70 and apoptosis markers compared to those within the sac, suggesting a partial protective effect of the cocoons. Furthermore, differences in biomarker sensitivity were observed across all the developmental stages and increased with prolonged exposure. These findings contribute to the understanding of EMF-induced biological effects in invertebrates and support the use of *P. tepidariorum* as a model species for environmental electromagnetic pollution.

## 1. Introduction

Electromagnetic fields (EMFs) are an often-overlooked abiotic environmental factors that originate from various anthropogenic sources, including mobile phones, digital enhanced cordless telecommunications (DECT) phones, Bluetooth devices, base stations, Wi-Fi networks, 4G and 5G infrastructure, power lines, and virtually all electrical appliances. The World Health Organization (WHO) has classified a radio frequency electromagnetic field as a potentially carcinogenic agent (Group 2B), which has fueled growing scientific and societal concerns regarding its potential effects on human health and the environment [1]. Despite an increasing body of research, the precise mechanisms through which EMFs interact with biological systems remain poorly understood and require further systematic investigation.

Recent studies have highlighted the potential impact of EMFs not only on human health but also on invertebrates. Invertebrate species, which are critical components of ecosystems, may exhibit physiological, behavioral, and cellular changes when exposed to electromagnetic fields [2,3]. The effects vary depending on the frequency, intensity, and duration of exposure.

Frequencies in the range of 300 Hz to 10 MHz are prevalent in various electronic devices and communication systems, including industrial equipment and certain medical devices. Health Canada has highlighted that EMFs within this frequency range can induce internal electric fields in biological tissues, potentially affecting excitable tissues such as nerves and muscles [4]. Additionally, the Health Council of the Netherlands has reviewed studies indicating that exposure to EMFs up to 10 MHz may lead to biological effects, such as changes in nerve and muscle excitability [5]. The 10 MHz frequency was selected based on previous reports indicating biological sensitivity in this range. Notably, it is close to 13.56 MHz, a frequency widely used in Near Field Communication (NFC) technology in modern smartphones, which enhances the environmental relevance of our study.

The implementation of fifth-generation (5G) wireless communication is expected to further escalate EMF exposure by increasing the number of base stations and high-frequency electromagnetic emitters. While EMFs have always been present in human environments, the intensity and ubiquity of artificial sources have reached unprecedented levels. Understanding how EMFs interact with biological systems, particularly at the molecular level, is a crucial research priority. Electromagnetic fields (EMFs) can affect biological systems on multiple levels, ranging from physiological and behavioral alterations to cellular and subcellular changes [2,3]. However, while whole-organism effects may take longer to manifest, cellular stress responses can provide sensitive and timely indicators of EMF-induced biological impact. Understanding these early changes is, therefore, crucial for identifying potential risks and mechanisms of action before more complex systemic damage develops.

Heat shock proteins (HSPs), particularly HSP70, play a crucial role in cellular stress responses. These proteins assist in maintaining protein homeostasis by preventing misfolding and aggregation, thereby promoting cell survival under adverse conditions [6]. Elevated HSP70 expression has been reported following EMF exposure in various organisms [7,8]. The upregulation of HSP70 in response to EMFs suggests a compensatory mechanism to mitigate potential protein damage, although excessive activation may indicate cellular distress [9,10,11]. Although the minority of studies focus exclusively on invertebrates, the pattern of HSP upregulation appears consistent across multiple species, supporting the hypothesis that EMFs are perceived as environmental stressors at the cellular level [12].

Apoptosis, or programmed cell death, is a key process that maintains cellular homeostasis and eliminates cells damaged both by natural developmental processes and by exposure to various environmental stressors. The EMF as a factor potentially modifying this process is insufficiently understood. A comprehensive literature review focusing on this topic presents investigations suggesting the sensitivity of apoptosis to EMF of various parameters used in the experiments [13]. At the same time, it also identifies exposure results that do not support such a conclusion [13]. Unrepaired DNA damage, oxidative stress, and disrupted cellular signaling pathways may trigger apoptotic cascades, ultimately affecting tissue integrity and organismal health [14].

Recent studies have shown that in invertebrate models such as *D. melanogaster*, *Caenorhabditis elegans*, and mollusks like *Mytilus galloprovincialis*, EMF exposure has been associated with increased apoptotic activity, suggesting a conserved stress response mechanism across taxa [15]. For instance, in *Drosophila* sp., the EMF exposure led to altered expression of apoptotic genes such as reaper and hid, and increased DNA fragmentation in neural and reproductive tissues [16]. Similarly, in bivalve mollusks, exposure to ELF-EMFs resulted in increased levels of caspase-3 activity, an essential executor of apoptosis [17]. These findings indicate that EMFs may act as environmental stressors capable of disrupting cellular homeostasis in invertebrates, triggering apoptosis.

In the above-mentioned works, the duration of experimental exposure of various invertebrates to EMF varied greatly, from several to several dozen minutes [16] to 10 days [11]. In turn, the cell lines were exposed for several dozen hours. Due to the small number of reference materials, two exposure durations were adopted in this study: 24 h and 72 h, to enable the assessment of both early (e.g., synthesis of stress proteins) and late (initiation of cell death processes) responses of cells to the electromagnetic field.

Spiders, particularly *P. tepidariorum*, present an advantageous model for studying the biological effects of EMFs. *P. tepidariorum* is a well-recognized species with a sequenced genome, facilitating molecular analyses [18]. Its laboratory-friendly rearing conditions further enhance its suitability as a model organism. Additionally, what is particularly important for this work, spider embryos are encased in a thin, single-layered chorion, offering minimal protection against environmental stressors [19,20]. Therefore, spiders at their embryonic stage may appear as indicators of EMF exposure and provide sensitive biomarkers of its harmful effects. This study will compare EMF-induced effects in embryos exposed within and outside the protective sac, providing insights into the shielding properties of biological barriers [21].

The choice of 10 MHz for electromagnetic field (EMF) exposure in this study is grounded in its environmental relevance due to technological applications, as a result of the wide range of frequencies the organisms are exposed to. Various findings underscore the importance of investigating the biological impacts of EMF exposure at 10 MHz, a frequency commonly encountered in both occupational and environmental settings.

Based on these considerations, in the present study, we made an attempt to assess the hazardous effects of 10 MHz EMF towards *P. tepidariorum*, a spider species commonly found in human-associated environments [6]. To achieve this goal, we focus on representative biochemical and cellular markers that indicate stress responses and cellular damage following EMF exposure. The markers examined in this study include the changes in the levels of heat shock proteins (HSPs) and apoptotic indicators, i.e., the percentages of living and apoptotic cells. These biomarkers offer insights into how EMFs may affect cellular homeostasis and stress adaptation mechanisms in invertebrates.

Studies on the effects of invertebrate exposure to EMF are still scarce, leaving a large knowledge gap. They are still random and preliminary. However, in order to assess the effects of exposure to EMF as comprehensively as possible in this study, an attempt was made to assess them at various developmental stages of spiders. Each development stage has its own specificity, and a different defense strategy may be launched at different stages depending on energy priorities. Therefore, the aim of this study was to test the following hypotheses using *P. tepidariorum* at various developmental stages.

1. Due to the protective potential of the cocoon, identified in its defense against pathogens and physical factors (e.g., humidity, temperature), it also serves as a protective barrier against EMF. Protective effectiveness of cocoon coverings is manifested by significantly lower rates of stress and/or damage (apoptosis parameters, HSP70 levels) measured in cocoon-protected embryos compared to unprotected ones.
2. Due to the specificity of different developmental stages, defensive mechanisms and sensitivity to EMF will vary. Therefore, the EMF exposure will cause differences in biomarker levels between exposed and non-exposed individuals.
3. There are developmental stages where the sensitivity of biomarkers is dependent on the duration of EMF exposure. As exposure time increases, the levels of selected markers elevate.

## 2. Results

### 2.1. Factor of Exposure

The experiment evaluated the effect of electromagnetic field exposure on the viability of spider cells across different developmental stages—egg (with and without a cocoon), young and adult spiders. The percentage of live, non-apoptotic cells was measured after 24 and 72 h using the ELISA apoptosis assay.

#### 2.1.1. Apoptosis—Percentage of Live Cells

**Figure 1:**
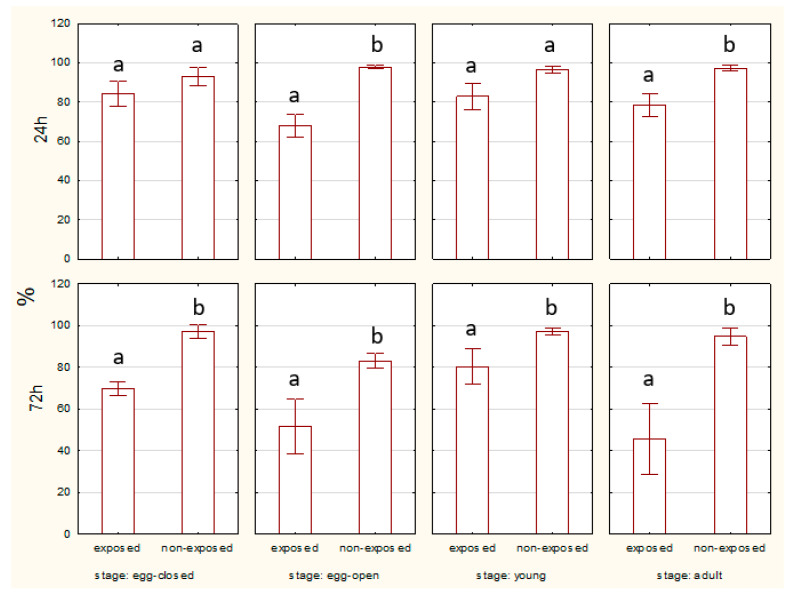
Percentage of living cells of spiders at various life stages, exposed or non-exposed to 10 MHz in relation to exposure duration. a, b—different letters denote statistically significant differences between exposed and non-exposed groups within stage; ANOVA *p* ≤ 0.05, Tukey test.

At the 24 h time point, no statistically significant difference was observed in the percentage of live, non-apoptotic cells between exposed and non-exposed groups of eggs with or without a cocoon. However, at the 72 h time point, significant differences were evident. Non-exposed eggs exhibited a higher percentage of live cells compared to the exposed groups. After 24 h, there was no significant difference in live cell percentage between the exposed and non-exposed groups. However, after 72 h, non-exposed young spiders showed a significantly higher percentage of live cells compared to the exposed group. The percentage of live cells remained consistently higher in non-exposed adults compared to exposed ones for both 24 and 72 h. Statistically significant differences were observed after 72 h of exposure (Figure 1).

**Figure 2:**
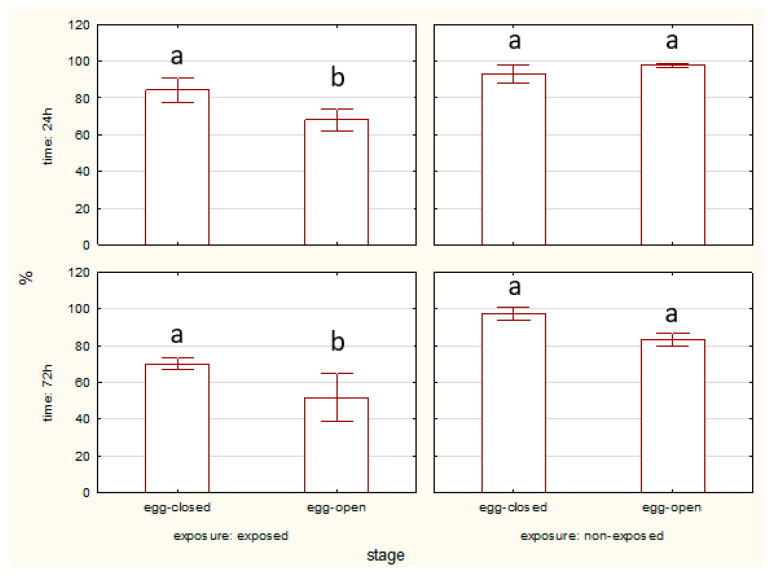
Percentage of living cells of spider eggs with cocoons (egg-closed) and without cocoons (egg-open) exposed or non-exposed to 10 MHz in relation to exposure duration (24 h and 72 h); a, b—different letters denote statistically significant differences between egg-closed and egg-open groups; ANOVA *p* ≤ 0.05, Tukey test.

The percentage of living midgut cells was higher in egg-closed stages than in egg-open stages after 24 and 72 h of EMF exposure. No significant differences between egg-closed and egg-open stages were observed in the non-exposed groups (Figure 2).

#### 2.1.2. Apoptosis—Percentage of Apoptotic Cells

Across all developmental stages, exposed individuals consistently exhibit higher apoptotic cell counts compared to non-exposed controls.

**Figure 3:**
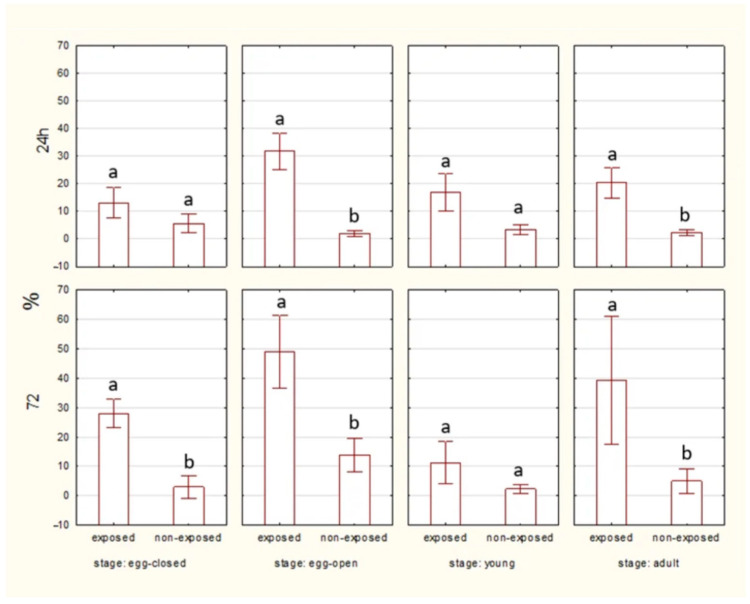
Percentage of total apoptotic cells of spiders at various life stages, exposed or non-exposed to 10 MHz in relation to exposure duration. a, b—different letters denote statistically significant differences between exposed and non-exposed groups within stage; ANOVA *p* ≤ 0.05, Tukey test.

Significant differences seen between exposed and non-exposed groups are present in specific stages and time points. Figure 3 presents the total number of apoptotic cells in different developmental stages of spiders (egg-closed, egg-open, young, and adult) after 24 and 72 h of exposure to an EMF, compared to a non-exposed control group. In the egg-closed stage, no significant difference is observed between exposed and non-exposed groups at the 24 h time point, but at the 72 h time point, the exposed group shows significantly higher apoptosis than the non-exposed group (*p* < 0.05), indicating a cumulative effect of EMF exposure over time. In the egg-open stage and adults, at both 24 and 72 h time points, exposed individuals exhibit significantly higher apoptosis than non-exposed individuals (*p* < 0.05). In the young stage, no significant differences are observed between exposed and non-exposed individuals at any time point, suggesting a relative resistance to EMF-induced apoptosis in this developmental stage. EMF exposure significantly increases apoptosis, particularly in the egg-open and adult stages, with the effect being more pronounced over time (72 h).

**Figure 4:**
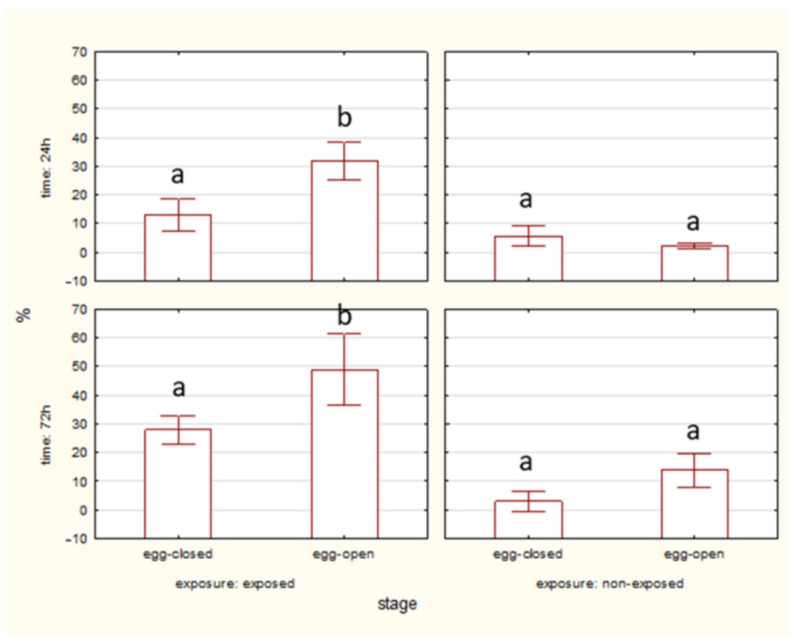
Percentage of total apoptotic cells of spider eggs with cocoons (egg-closed) and without cocoons (egg-open) exposed or non-exposed to 10 MHz in relation to exposure duration (24 h and 72 h); a, b—different letters denote statistically significant differences between egg-closed and egg-open groups; ANOVA *p* ≤ 0.05, Tukey test.

The percentage of apoptotic cells was significantly higher in egg-open stages compared to egg-closed stages after 24 and 72 h of EMF exposure. No significant differences between stages were observed in the non-exposed groups (Figure 4).

**Figure 5:**
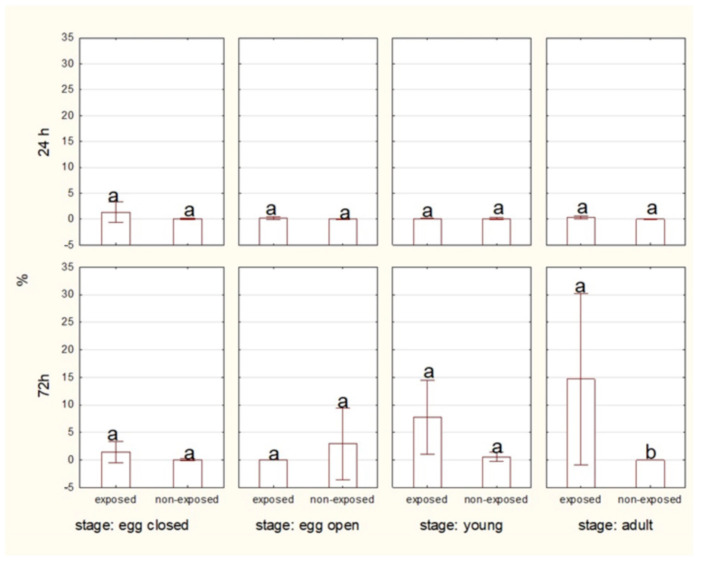
Percentage of dead cells of spiders at various life stages, exposed or non-exposed to 10 MHz in relation to exposure duration. a, b—different letters denote statistically significant differences between exposed and non-exposed groups within stage; ANOVA *p* ≤ 0.05, Tukey test.

Over 24 h of exposure, there were no significant differences in the percentage of dead cells between exposed and non-exposed groups. However, in the adult stage, a higher level of dead cells was observed after 72 h of 10 MHz exposure (Figure 5).

#### 2.1.3. HSP70 Level

**Figure 6:**
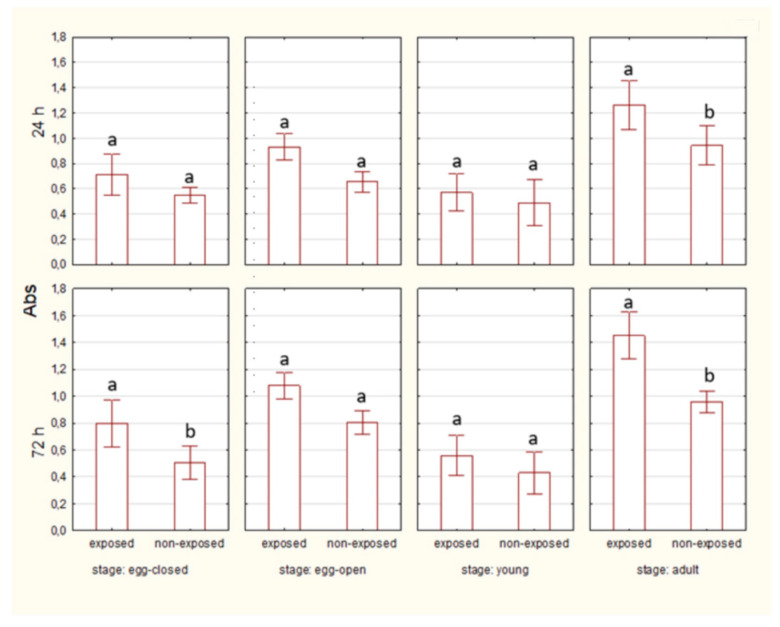
The level of HSP70 in cells of spiders at various life stages, exposed or non-exposed to 10 MHz in relation to exposure duration. a, b—different letters denote statistically significant differences between exposed and non-exposed groups within stage; ANOVA ≤ 0,05, Tukey test.

After 24 h of exposure, HSP70 levels are visibly higher in the exposed groups than in the non-exposed groups only in the case of adult spiders. By 72 h, HSP70 levels remain elevated in the exposed groups in egg-closed and adult spiders. (Figure 6).

**Figure 7:**
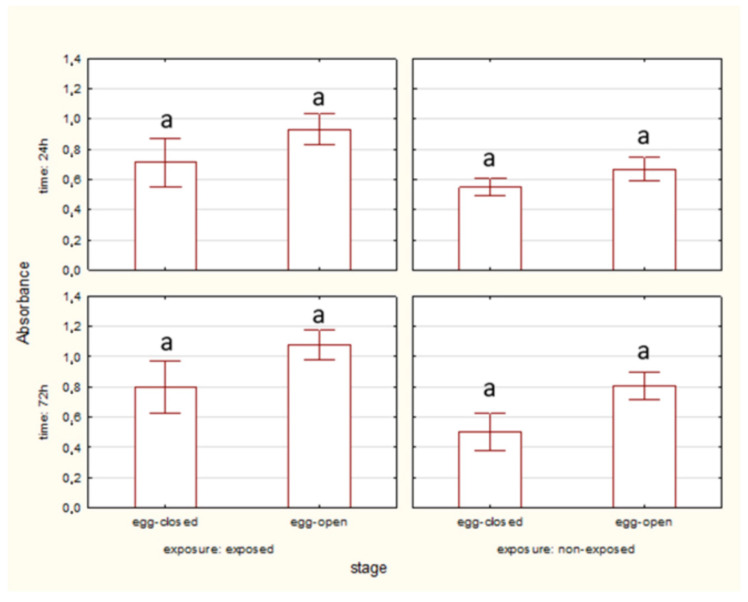
The level of HSP70 in cells of spider eggs with cocoons (egg-closed) and without cocoons (egg-open) exposed or non-exposed to 10 MHz in relation to exposure duration (24 h and 72 h); a, b—different letters denote statistically significant differences between egg-closed and egg-open groups; ANOVA *p* ≤ 0.05, Tukey test.

No significant differences in HSP70 level were observed between egg-closed and egg-open stages after 24 and 72 h, both in exposed and non-exposed groups (Figure 7).

### 2.2. Factor of Stage

#### 2.2.1. Apoptosis—Percentage of Live Cells

**Figure 8:**
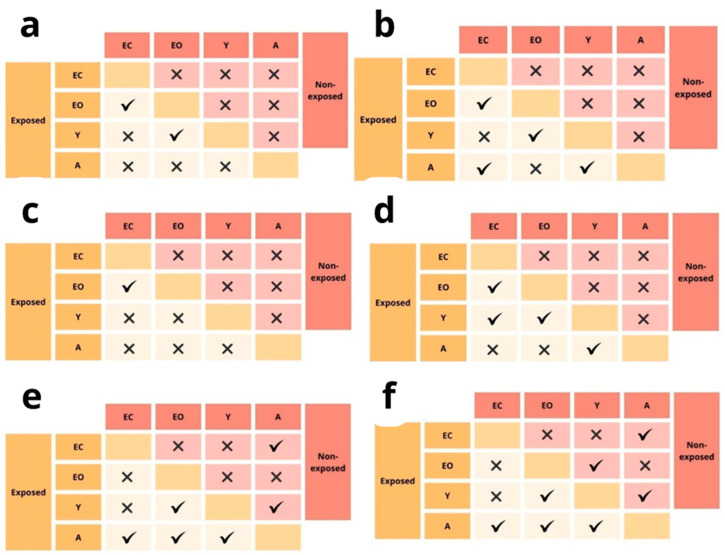
The results of Tukey test (ANOVA) for: differences between the percentage of live cells of spiders exposed or non-exposed to 10 MHz for 24 h (**a**) and 72 h (**b**), differences between the percentage of total apoptotic cells of spiders exposed or non-exposed to EMF for 24 h (**c**) and 72 h (**d**) and differences between the percentage of the level of HSP70 in midgut cells of spiders exposed or non-exposed to EMF for 24 h (**e**) and 72 h (**f**) in relation to the developmental stage; √—significant difference, x—insignificant difference (*p* ≤ 0.05), yellow (headings) and pale pink (significance marking) area—exposed, red (headings) and pink (significance marking) area—non-exposed, EC—egg-closed, EO—eggs-open, Y—young, A—adult.

In the exposed group, significant differences were observed among developmental stages. The egg-open (EO) group exhibited a significantly lower percentage of live cells compared to the egg-closed (EC) group (*p* < 0.05). The viability in young (Y) spiders was comparable to egg-closed individuals, whereas the adult (A) stage showed intermediate values. In the non-exposed condition, no statistically significant differences were detected between groups, indicating that under normal conditions, developmental stage does not affect cell viability (Figure 8a,b).

A more pronounced effect was observed after 72 h of exposure. In the exposed group, the percentage of live cells in egg-open individuals remained significantly lower than in egg-closed individuals (*p* < 0.05). Interestingly, young spiders also showed reduced viability compared to “egg-closed” individuals, whereas adults displayed values comparable to other groups (Figure 8a,b).

In contrast, in the non-exposed condition, no statistically significant differences were observed between any developmental stages, confirming that apoptosis levels remain stable in the absence of EMF exposure. However, in the absence of exposure, no stage-specific differences in cell viability were observed (Figure 8a,b).

#### 2.2.2. Apoptosis—Percentage of Total Apoptotic Cells

After 24 h of exposure, the egg-closed (ec) and egg-open (eo) stages differ significantly (*p* < 0.05), with a higher number of apoptotic cells in egg-open individuals. The young and adult stages do not differ significantly from each other or from egg-closed, indicating a moderate apoptotic response across these stages. After 72 h, a significant increase in apoptosis is observed in the egg-open group compared to all other stages (*p* < 0.05). The adult group shows an intermediate response, significantly differing from both the egg-open and young stages. Egg-closed and young stages do not differ significantly. In the non-exposed condition, no significant differences in apoptotic cell count are observed across developmental stages or time points (Figure 8c,d).

#### 2.2.3. HSP70 Level

After 24 h, the exposed groups exhibit statistically significant increases in HSP70 levels relative to controls, with the egg-open and adult stages showing the greatest differences. By 72 h, these differences become even more pronounced; the egg-open stage, in particular, displays a statistically significant elevation in HSP70 compared to both non-exposed spiders and the other developmental stages. In contrast, HSP70 levels in non-exposed groups remain relatively stable over time (Figure 8e,f).

### 2.3. Factor of Time

The egg-open and adult stage showed a significant decrease in cell viability after 72 h of exposure (*p* < 0.05, Table 1). For egg-closed stage and young spiders, no significant differences were observed between the 24 and 72 h time points, implying that this developmental stage does not experience increased sensitivity over time. Unlike, in the egg closed and young spiders, cell viability remained stable between time points. In the non-exposed condition, no statistically significant differences were observed between 24 and 72 h in any developmental stage.

Whereas, the total number of apoptotic cells in spiders at different developmental stages, egg-open spiders exhibited a statistically significant increase in total apoptotic cells at the 72 h time point compared to the 24 h time point. Similarly, adult spiders also showed a significant rise in apoptotic cell count at 72 h relative to 24 h. In contrast, egg-closed and young spiders did not display significant differences between the two time points. Furthermore, in the non-exposed controls, no significant changes in apoptotic cell numbers were observed between 24 and 72 h.

The ELISA test measuring HSP70 levels in spider cells showed no significant differences between the 24 h and 72 h time points across all groups.

#### Correlations Between Parameters

Correlation analyses revealed significant relationships between HSP70 levels and the percentage of total apoptotic cells in spider samples in pooled analysis and when each factor was analyzed separately. The only non-significant correlation was observed in non-exposed individuals (Table 2).

## 3. Discussion

The findings presented here align with existing research indicating that EMF exposure can modulate HSP70 levels, thereby influencing stress responses and regenerative processes.

One such study by Vilić et al. (2024) [22] investigated the impact of radiofrequency (RF) EMFs on the honeybee (*Apis mellifera*). In this study, honeybee workers were exposed to a 900 MHz RF-EMF, simulating the frequencies commonly emitted by mobile phone base stations. The exposure duration varied between 10 and 30 min, with different power densities to assess dose-dependent effects. The results indicated a significant upregulation of HSP70 in the brain tissue of exposed bees compared to the control group.

The authors hypothesized that the increase in HSP70 levels might be linked to oxidative stress, given that previous research has established a connection between RF-EMFs and the generation of reactive oxygen species (ROS) [22]. Similar findings have been observed in freshwater planarians (*Dugesia dorotocephala*), where exposure to 60 Hz ELF-EMFs also resulted in increased HSP70 levels and enhanced regenerative abilities (Goodman et al., 2009) [23]. While the frequency and type of EMF differed in these studies, both suggest that HSP70 acts as a universal stress response protein in invertebrates, modulating cellular defense mechanisms.

Our findings, which indicate that non-ionizing EMF exposure leads to an increase in HSP70 levels in spiders, align with these previous studies on bees and planarians. Given that HSP70 plays a crucial role in cellular protection and protein homeostasis under stress conditions, its induction under EMF exposure might be an adaptive mechanism across different invertebrate species. However, variations in frequency, power density, and exposure duration could lead to species-specific differences in stress response dynamics.

The study by Wyszkowska et al. (2016) [24] provides significant evidence that exposure to ELF EMFs (above 4 mT) induces notable physiological and behavioral changes in the desert locust (*Schistocerca gregaria*), including an increase in HSP70 levels. The upregulation of HSP70 suggests that ELF-EMFs trigger cellular stress pathways in locusts, likely as a protective mechanism against EMF-induced physiological disruptions [24]. In the context of this research on spiders, the study by Wyszkowska et al. (2016) [24] provides a valuable reference point. Our findings similarly indicate that non-ionizing EMFs lead to increased HSP70 expression, reinforcing the idea that invertebrates recognize EMFs as a stressor and respond through conserved molecular pathways. However, while Wyszkowska et al. (2016) [24] focused on ELF-EMFs and their effects on neuromuscular activity in locusts, this research explores how different EMF frequencies influence stress-protein responses in arachnids. Given HSP70’s role as a molecular chaperone facilitating protein folding and protection against stress-induced damage, its induction may serve as a protective mechanism to mitigate EMF-induced cellular stress.

The impact of electromagnetic fields (EMFs) on apoptosis has also been a subject of scientific investigation. Manta et al. (2016) [25] provide significant evidence that RF EMF can induce apoptosis in *D. melanogaster*, further supporting findings from other invertebrate studies. The researchers exposed *D. melanogaster* to 1.8 GHz radiofrequency radiation for varying durations (up to 6 h per day) and measured changes in reproductive function, oxidative stress markers, and apoptotic activity. Their results revealed an upregulation of apoptotic processes in reproductive tissues, as demonstrated by increased caspase-3 activity and TUNEL staining, which marks DNA fragmentation in apoptotic cells. These findings suggest that prolonged RF EMF exposure induces cellular stress, leading to apoptosis. The findings align with our results, which suggest that non-ionizing EMFs significantly impact apoptotic pathways in spiders, strengthening the argument that non-ionizing EMFs, particularly at frequencies relevant to telecommunications, have a measurable impact on apoptosis in invertebrates. If EMF exposure consistently induces apoptosis in invertebrates, it may contribute to disruptions in population dynamics. Further research should focus on long-term effects, potential adaptive responses, and species-specific sensitivities to EMF exposure [25].

A study by Sagioglou et al. (2016) [26] examined the impact of various EMF sources on *D. melanogaster* and *D. virilis*, focusing on apoptosis during oogenesis. The researchers exposed newly emerged female flies to different EMF sources, including mobile phones, DECT handsets, Wi-Fi networks, Bluetooth, and microwave ovens, for 3–7 days. They observed a significant increase in apoptotic cell death in ovarian follicles across all exposure groups compared to controls. Interestingly, no cumulative effect was noted when exposure duration was increased or when different EMF sources were used sequentially. These findings suggest that EMFs, even at low intensity levels, can induce apoptosis in the reproductive system of Drosophila, highlighting the potential impact of EMF exposure on invertebrate health. These findings support the results of the present study, where exposure to non-ionizing EMFs influenced apoptotic activity in spiders. The consistency between different invertebrate models suggests that the impact of EMFs on apoptosis may be a widespread phenomenon rather than a species-specific effect. However, further research is necessary to determine whether these effects are reversible, dose-dependent, or influenced by additional environmental factors [26].

In our research, across all developmental stages, exposed individuals consistently exhibit higher apoptotic cell counts compared to non-exposed controls, confirming the pro-apoptotic effect of EMF exposure. In the adult stage, at the 72 h time points, the exposed group shows a significant increase in apoptosis (*p* < 0.05). This suggests a delayed apoptotic response to EMF exposure in adults, potentially due to differences in cellular repair mechanisms or slower accumulation of damage. The non-exposed control group shows consistently low apoptosis, confirming that the observed effects result from EMF exposure rather than natural cell death processes.

The study by Agrawal et al. (2021) [27] demonstrates that ELF-EMF can differentially affect various developmental stages of *D. melanogaster*. Exposure to ELF-EMF (75 Hz, 550 µT) under both acute and chronic conditions led to delayed development, reduced survival, impaired locomotion, cellular damage, and elevated oxidative stress. Interestingly, the effects varied depending on the stage of exposure—larvae were more sensitive to locomotor impairment and gut cell damage, while adults showed subtler neuromuscular changes. These findings suggest that the sensitivity to EMF may be stage-specific, possibly due to shifting physiological priorities such as energy allocation, development, and protection of genetic material. This supports the hypothesis that electromagnetic fields may interfere differently across ontogeny, with certain stages being more vulnerable due to less developed protective mechanisms or heightened metabolic demand. The study suggests that ELF-EMFs can act as a physiological stressor, particularly during early developmental stages [26,27].

In our research, the egg-open stage showed a significant decrease in live cell viability after 72 h of exposure (*p* < 0.05), suggesting that prolonged exposure to EMF has a cumulative negative effect when the spider cocoon is absent. These findings highlight the vulnerability of egg-open individuals to prolonged EMF exposure, as their viability decreases significantly over time. However, egg-closed, young, and adult stages exhibit stable viability, suggesting a protective effect of the cocoon or increased resistance to EMF-induced apoptosis. The absence of viability changes in the non-exposed group further confirms that the observed differences are specifically induced by electromagnetic field exposure. These findings highlight the vulnerability of egg-open and adult individuals to prolonged EMF exposure, as their viability decreases significantly over time. However, egg-closed and young stages exhibit stable viability, suggesting a protective effect of the cocoon or increased resistance to EMF-induced apoptosis. The absence of viability changes in the non-exposed group further confirms that the observed differences are specifically induced by electromagnetic field exposure.

The more pronounced cellular response observed in adult spiders compared to young spiders may be attributed to differences in the maturity and efficiency of their defense mechanisms. Adult individuals possess fully developed cellular stress-response pathways, which may enable a more effective reaction to EMF exposure. In particular, adults may actively counteract EMF-induced damage through apoptosis, thereby limiting the accumulation of potentially harmful mutations. Additionally, the induction of HSPs in adults suggests the presence of a functional and responsive molecular chaperone system that contributes to cellular protection under stress conditions. Also in the study by Babczyńska et al. (2024) [28], interesting age-dependent differences were found in house crickets exposed to graphene oxide nanoparticles as an environmental stressor. Under optimal conditions, HSP70 and apoptotic pathways are interconnected, with the role of heat shock proteins as modulating and controlling factors at various stages of apoptotic pathways [28]. In contrast, young spiders, being in an active developmental stage, may prioritize energy allocation toward growth, differentiation, and basic physiological maintenance rather than toward costly cellular defense responses. At this stage of development, available energetic resources are limited and are likely directed toward processes essential for survival and development. Consequently, young may rely on different or less energy-intensive protective strategies, or may exhibit a reduced capacity to mount a detectable stress response at the cellular level. Moreover, EMF exposure represents a stressor that is not inherently integrated into the species’ evolutionary developmental trajectory. It is therefore possible that, during early developmental stages, organisms are less capable of redirecting defensive mechanisms toward a novel and evolutionarily unfamiliar stressor. As a result, the absence or attenuation of cellular stress markers in young spiders may reflect developmental constraints rather than a true lack of EMF sensitivity.

A significant correlation between HSP70 levels and apoptosis observed exclusively in EMF-exposed groups indicates that EMF exposure activates multiple cellular stress pathways in parallel. This relationship most likely reflects a common upstream stress signal induced by EMF, such as oxidative stress, disruption of protein homeostasis, and damage to cellular structures. These processes are known to simultaneously stimulate the expression of heat shock proteins as part of a proteotoxic stress response and activate apoptotic pathways when cellular damage exceeds adaptive capacity.

Under physiological conditions, HSP70 expression and apoptosis are regulated independently and remain at baseline levels. Therefore, the observed coupling of HSP70 induction and apoptosis appears to be a stress-specific response triggered by EMF exposure rather than a direct causal interaction between these two processes. As this study is exploratory, further investigations are required to determine the relative contribution of apoptosis versus other forms of cell death, including necrosis, and to clarify the primary molecular mechanisms underlying EMF-induced cellular stress.

Under stress conditions, apoptosis is a protective process aimed at minimizing side effects at higher levels of biological organization and restoring energy resources. In such circumstances, the inhibitory effects of HSPs are no longer significant. It should also be emphasized that both processes are stimulated by oxidative stress, which develops as a result of exposure to stress factors. HSPs are protective proteins whose synthesis is initiated, among other things, by oxidative stress [29,30].

Similar phenomena occur in the case of stress related to strong environmental pollution and in conditions of immunostimulation, which is also associated with the intensification of metabolic processes [31].

However, it should be emphasized that the activation of apoptosis does not exclude the involvement of other forms of cell death. The potential contribution of necrotic processes cannot be ruled out and should be addressed in future studies, as distinguishing between apoptosis and necrosis would provide a more comprehensive understanding of the cellular consequences of EMF exposure.

Importantly, this study should be considered a pilot investigation in the context of EMF-related research on arachnids. Given the limited existing data in this field, we focused on broadly applicable and well-established biomarkers of cellular stress to determine whether EMF exposure induces any measurable biological response at all. The use of universal biomarkers was intended to provide an initial indication of cellular disturbance rather than a complete characterization of all underlying mechanisms.

## 4. Materials and Methods

### 4.1. Model Animal

*Parasteatoda tepidariorum*, commonly known as the common house spider, is a small arachnid species characterized by a brownish coloration and distinctive markings on the abdomen. This species is frequently found in human dwellings, where it is considered beneficial due to its role in natural pest control. *P. tepidariorum* has a well-defined life cycle, making it a suitable model organism for developmental and environmental studies. After egg deposition, embryos develop within a silken egg sac (cocoon) for approximately 7–10 days, depending on environmental conditions such as temperature and humidity. Upon hatching, young spiders (spiderlings) undergo a series of molts over the course of several weeks to months, gradually maturing into adults. The time from egg to sexual maturity typically ranges from 30 to 60 days under laboratory conditions. Adult spiders can live for several months, with females often outliving males. This predictable and relatively short life cycle, along with its manageable size and ease of laboratory maintenance, makes *P. tepidariorum* an effective model for studying the effects of environmental factors such as electromagnetic fields at various developmental stages [32]. Spiders used in this experiment were randomly selected from a long-term breeding run in the authors’ institute. Spiders were kept in individual plastic containers under conditions of 25 ± 1 °C, 70% relative humidity, and a photoperiod of L:D—16 h:8 h.

In the described experiment, eggs or midgut cells in the case of young and adult *P. tepidariorum* were used as the biological material for assessing cellular responses to electromagnetic field (EMF) exposure. The midgut gland, often referred to as the hepatopancreas in invertebrates, serves as a central organ for detoxification and metabolic regulation. It plays a pivotal role in processing xenobiotics and managing oxidative stress, making it an ideal tissue for studying environmental stress responses [33,34].

### 4.2. Experimental Groups

Spiders were divided into four main groups: adult spiders, young spiders, eggs in cocoons and eggs without cocoons’ protection. Next, these groups were either exposed or not exposed to 10 MHz EMF models for 24 h and 72 h (Figure 8).

### 4.3. EMF Exposition

**Figure 9:**
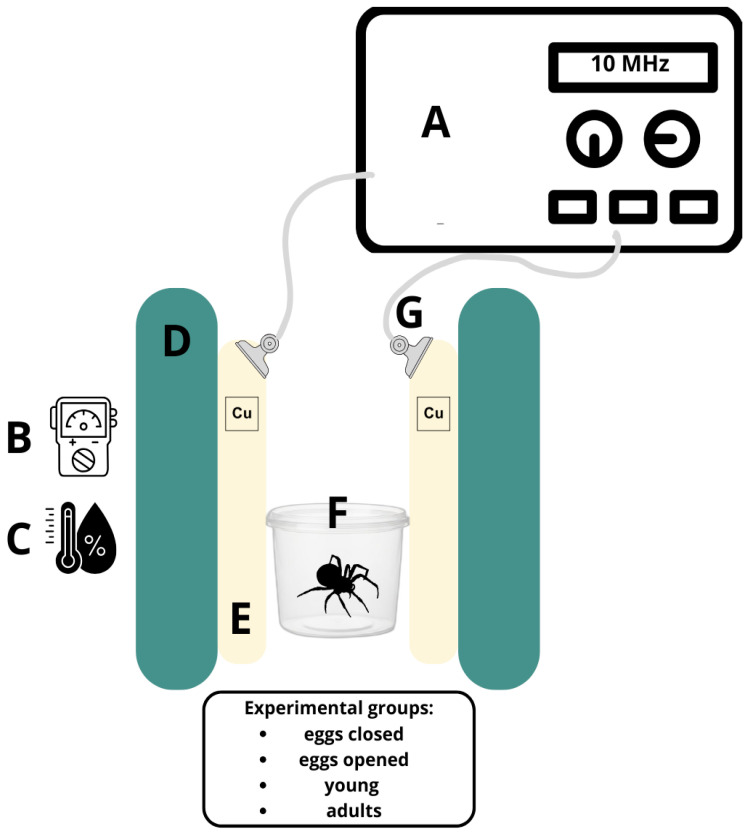
Experimental setup: A—EMF generator, B—intensity meter, C—hygrometer, D—rack, E—copper plate, F—plastic container with spiders inside, G—clip and cable.

During the exposure, thermal and humidity conditions were the same as in the rearing conditions. The experimental setup for electromagnetic field (EMF) exposure included a pair of parallel copper plates (10 cm × 10 cm) separated by a distance of 10 cm and connected to a sinusoidal signal generator (Funktionsgenerator 250350—Frederiksen Scientific, Olgod, Denmark), providing a frequency of 10 MHz. The electromagnetic field strength was monitored using a broadband field meter (Tenmars TM-196, Taipei, Taiwan). Samples were placed precisely in the central part of the experimental setup (Figure 9).

Given the dimensions of the setup, the field between the plates was in the near-field regime. The dominant component of the field in our conditions is the electric field, which was directly measured and was within the range of 4.264–5.045 V/m. Measurements were performed at multiple positions between the plates to verify the spatial distribution and approximate uniformity of the generated field.

Background magnetic field levels were monitored prior to exposure using a low-frequency range meter (Tenmars TM-191, Taipei, Taiwan), and ranged from approximately 0.12 µT. These values refer to low-frequency environmental background, mainly associated with power-frequency fields (50 Hz), and are not directly comparable with the high-frequency electromagnetic field generated in the present experimental setup. Additionally, the background field was measured by means of the Tenmars TM-196 m. These values for the electric component were equal to 0.2–0.5 V/m, which are typical for indoor RF background (WiFi/GSM).

An oscilloscope (Rigol DS1054Z, Suzhou, China) was also employed to ensure the stability and waveform characteristics of the generated signal throughout the exposure period.

### 4.4. Investigated Parameters and Tests

#### 4.4.1. HSP70

The concentrations of HSP70 were measured using an Enzyme-Linked Immunosorbent Assay (ELISA) following a standard protocol. Homogenates were prepared to contain 100 µg of protein in 100 µL and transferred into the wells of a 96-well microplate. The plate was incubated overnight at 4 °C to allow proteins in the samples to adsorb onto the well surfaces. After incubation, the wells were washed four times with PBS containing 0.1 M Tween-20 to eliminate unbound antigens. To block non-specific binding, 100 µL of 5% albumin solution was added to each well, followed by a 1 h incubation at 37 °C. Plates were then incubated for 2 h at 37 °C with primary antibody specific to the target protein: mouse monoclonal anti-HSP70 (Sigma-Aldrich, Merck KGaA, Darmstadt, Germany 1:1000). After additional washing, the secondary antibody, alkaline phosphatase-conjugated goat anti-mouse (Sigma-Aldrich, 1:1000), for HSP70, was applied. Following a 1 h incubation at 37 °C and final washes, a 10 mM p-nitrophenyl phosphate solution in diethanolamine buffer (pH 9.5) was added. This substrate reacts with the enzyme to produce a yellow product. After 30 min of incubation at room temperature, absorbance was read at 405 nm using a Tecan Infinite M200 microplate reader [35,36].

#### 4.4.2. Apoptosis

The Annexin V Muse Kit (Sigma-Aldrich/Millipore, Merck KGaA, Darmstadt, Germany) is a flow cytometry-based method used to detect and quantify apoptotic and necrotic cells in biological samples. This kit is particularly useful for studying programmed cell death and its mechanisms, such as in cancer research, immunology, and drug development.

The Annexin V Muse Kit uses Annexin V conjugated to a fluorescent marker (typically FITC or PE) to detect phosphatidylserine (PS) exposure on the outer membrane of cells. During apoptosis, the normally inner membrane of phosphatidylserine flips to the outer membrane, which is a key signal of early apoptosis. This kit uses Annexin V, which has a high affinity for PS, to bind and mark apoptotic cells. Propidium iodide (PI), a DNA-binding dye, is also included in the kit to differentiate between live, apoptotic, and necrotic cells. Live cells will exclude PI, apoptotic cells will be Annexin V-positive and PI-negative, and necrotic cells will be positive for both Annexin V and PI due to compromised cell membranes [37,38,39]. The kit is dedicated to the device: Muse^®^ Cell Analyzer (Millipore, Billerica, MA, USA), factory-equipped with appropriate software, which was used to measure apoptotic parameters with number of events: 2000–3000.

#### 4.4.3. Statistical Analysis

Prior to conducting ANOVA, the assumptions were examined. Normality of residuals was assessed using the Shapiro–Wilk test, and homogeneity of variances using Levene’s test. One-way ANOVA was then performed, followed by Tukey’s post hoc test (*p* < 0.05) to identify pairwise differences between groups. Pearson’s correlation coefficient was used to perform the analysis of correlations between the HSP70 and percentage of total apoptotic cells. All analyses were conducted using STATISTICA (Dell Inc. Round Rock, TX, USA (2016). Dell Statistica (data analysis software system), version 13. software.dell.com). Number of replicates (n) for specific sample types were min. 6 for each stage.

## 5. Conclusions

These findings confirm that the individuals from the egg-open are the most susceptible to EMF-induced changes, especially after prolonged exposure. The presence of a cocoon (egg-closed) provides some protection, while early and adult stages exhibit relative resistance.

A spider cocoon seems to provide effective protection against EMF exposure, as evidenced by a higher number of living midgut cells and lower rates of apoptosis in cocoon-covered embryos. Although no significant differences were observed in HSP70 levels, it is possible that the exposure conditions triggered only moderate cellular stress, sufficient to induce apoptosis but not strong enough to activate a detectable heat shock protein response in eggs. This suggests that the cocoon acts as a physical barrier primarily mitigating direct cellular damage, rather than fully preventing the perception of environmental stress.

The findings suggest that the most sensitive stages to EMF exposure are the egg open and adult spider stages. This heightened sensitivity may be due to developmental and physiological factors unique to these stages. During the egg open stage, the organism’s cellular structures may be more vulnerable to external influences, such as EMF, due to ongoing growth and differentiation processes. These variations in sensitivity likely result from differences in developmental stages, where the defensive mechanisms and cellular responses are not yet fully established or are more active, making them more susceptible to environmental stressors like EMF.

Exposure duration affects mainly the individuals at the most EMF sensitive developmental stages (egg-open and adult spiders) as evidenced by increased percentage of total apoptotic cells and decrease in the number of living cells with longer EMF exposure. This progressive deterioration suggests that prolonged exposure at these stages may lead to stronger and more lasting effects on the exposed population. Such effects may lead to lower individual survival rates, developmental abnormalities, and reduced reproductive success. Over time, this may likely weaken the overall fitness of the population and contribute to its gradual decline under prolonged EMF exposure.

## Figures and Tables

**Table 1:** Statistically significant time-dependent differences in the percentage of live and apoptotic midgut cells of spiders exposed to 10 MHz; Tukey test, ANOVA (*p* ≤ 0.05).

Live	
Stage	Time
Eggs open exposed	24 h > 72 h
Adult exposed	24 h > 72 h
Apoptotic	
Eggs open exposed	24 h < 72 h
Adult exposed	24 h < 72 h

**Table 2:** Pearson’s correlation coefficients between HSP70 levels and percentage of total apoptotic cells in spider samples (whole eggs and young spider bodies and midgut glands of adult spiders) exposed or not exposed to 10 MHz; *p* ≤ 0.05.

	Correlation Coefficient
pooled	0.540840425
factor: age	
eggs (open)	0.79923423
eggs (closed)	0.643380416
young	0.431749956
adults	0.608002956
factor: time	
24 h	0.421922198
72 h	0.603164373
factor: exposure	
exposed	0.468106268
not exposed	Not significant

## Data Availability

The original contributions presented in this study are included in the article/Supplementary Material. Further inquiries can be directed to the corresponding author.

## Supplementary Materials

The following supporting information can be downloaded at: https://www.mdpi.com/article/10.3390/ijms27094088/s1.
