# Supplementary material for: Assessment of the Effects of Electromagnetic Fields on Apoptosis and Stress Protein Biomarkers in the Spider Parasteatoda tepidariorum
Source: Int J Mol Sci. 2026 May 2;27(9):4088. doi: 10.3390/ijms27094088 (PMC13164240; doi:10.3390/ijms27094088)
Supplement: Supplementary file 1 [file ijms-27-04088-s001.zip › ijms-4254170-supplementary.pdf]

Apoptosis			72h		
	live	early	late	dead	total
ZN	96,85	2,75	0	0,4	2,75
ZN	99,4	0,6	0	0	0,6
ZN	100	0	0	0	0
ZN	95,98	2,1	2,77	0	4,87
ZN	91,25	6,52	3,01	0	9,53
ZN	99,97	0,03	0	0	0,03
ZE	73,61	0,64	21,24	4,51	21,88
ZE	69,35	30,5	0,15	0	30,65
ZE	65,45	34,5	0,05	0	34,55
ZE	67,62	25,48	5,06	0,02	30,54
ZE	74,04	12,63	10,44	3,07	23,07
ZE	70,08	24,56	2,38	1,09	26,94
OE	59,3	40,4	0,3	0	40,7
OE	28,25	71,6	0,15	0	71,75
OE	60,55	39,35	0,1	0	39,45
OE	45,15	53,05	1,8	0	54,85
OE	61,22	29,09	15,26	0,1	44,35
OE	56,67	37,38	5,09	0	42,47
MN	97,4	2,35	0,05	2,4	2,4
MN	99,3	0,55	0,1	0,05	0,65
MN	98,3	1,1	0,3	0,3	1,4
MN	97,85	1,75	0,1	0,3	1,85
MN	94,55	5	0,15	0,3	5,15
MN	95,79	2,4	0,16	0,2	2,56
ME	74,3	3,25	9,65	12,9	12,9
ME	76,9	0,35	8,6	14,15	8,95
ME	80,2	0,3	4,85	14,65	5,15
ME	96,95	1,1	0,75	1,2	1,85
ME	78,98	10,97	9,22	1,5	20,19
ME	74,29	8,82	9,87	2,35	18,69
DE	23,1	55,02	12,5	10	67,52
DE	47,98	8,56	13,04	29,03	21,6
DE	55,82	13,4	20,08	10,12	33,48
DE	34,17	60,06	5,54	0	65,6
DE	42,09	5,45	14,36	38,2	19,81
DE	72,11	24,78	2,3	0,7	27,08
DN	91,92	8,08	0	0	8,08
DN	88,03	6,57	5,4	0	11,97
DN	99,2	0,8	0	0	0,8
DN	96,4	3,51	0,05	0,04	3,56
DN	97,28	2,1	0,02	0	2,12
DN	96,93	3,2	0,06	0	3,26
ON	88	11,75	0,25	0	12
ON	82,6	17,05	0,3	0,05	17,35
ON	85,65	14,3	0,05	0	14,35
ON	79,75	20,1	0,05	0	20,15

Apoptosis
ZN
ZN
ZN
ZN
ZN
ZN
ZN
ZE
ZE
ZE
ZE
ZE
ZE
OE
OE
OE
OE
OE
OE
MN
MN
MN
MN
MN
MN
ME
ME
ME
ME
ME
ME
DE
DE
DE
DE
DE
DE
DN
DN
DN
DN
DN
DN
ON
ON
ON

ON	80,2	0,3	4,85	14,65	5,15	ON
						ON

live	early	24h<br>late	dead	total
88,61	5,7	5	0,3	10,7
92,15	7	0	0	7
98,01	0,8	1,2	0	2
86,25	7,75	5	0	12,75
99,05	0,67	0,03	0	0,7
95,26	3,25	0,23	0,1	3,48
91,33	2,2	3,08	0	5,28
73,3	12	9,8	5,2	21,8
86,02	3	10	0	13
88,09	6	4,7	0,3	10,7
90,2	4,6	3,2	2	7,8
88,2	4,4	3,66	0,1	8,06
79,79	10,2	7,12	0,6	17,32
76,85	22,53	1,12	0,2	23,65
66,02	19,54	10,45	0,6	29,99
59,55	29,45	7,6	0	37,05
65,56	15,52	26,57	0,3	42,09
71,84	12,56	16,95	0	29,51
68,34	7,97	21	0,2	28,97
97,75	2,15	0	0,1	2,15
95,35	4,5	0,15	0	4,65
94,15	5,75	0,1	0	5,85
97,25	2,7	0	0,5	2,7
96,37	3,3	0,67	0	3,97
98,37	1,2	0,06	0	1,26
82,35	17,5	0,05	0,1	17,55
71,7	27,65	0,5	0,15	28,15
79,95	19,65	0,25	0,15	19,9
88,5	11,45	0,05	0	11,5
91,2	8,8	0	0	8,8
83,65	15,8	0,45	0,1	16,25
80,65	17,7	0,95	0,7	18,65
76,85	21,75	1	0,4	22,75
67,35	24,87	5,67	0,2	30,54
79,73	17,87	0,6	0	18,47
81,98	15,26	0,8	0,7	16,06
83,92	13,89	1,8	0	15,69
97,82	2,02	0	0	2,02
98,82	1,08	0	0	1,08
96,16	3,12	0,02	0	3,14
96,47	2,2	0,9	0	3,1
99,11	0,8	0	0	0,8
95,38	3,4	0,4	0,1	3,8
97,95	2,05	0	0	2,05
96,7	3,2	0,1	0	3,3
98,45	1,4	0,05	0,1	1,45

99,15	0,7	0,05	0,1	0,75
96,9	3,05	0,05	0	3,1
